# The complete mitochondrial genome of the semiterrestrial crab, *Chiromantes neglectum* (Eubrachyura: Grapsoidea: Sesarmidae)

**DOI:** 10.1080/23802359.2016.1186509

**Published:** 2016-07-08

**Authors:** Yuhui Xing, Xiaoping Ma, Yuqing Wei, Da Pan, Wenliang Liu, Hongying Sun

**Affiliations:** a Jiangsu Key Laboratory for Biodiversity and Biotechnology, College of Life Sciences, Nanjing Normal University, Nanjing, China;; b College of Life Sciences, Nanjing Normal University, Nanjing, China;; c Schoole of Ecological and Environmental Sciences, East China Normal University, Shanghai, China

**Keywords:** *Chiromantes neglectum*, eubrachyuran phylogeny, gene rearrangement, mitochondrial genome

## Abstract

The complete mitogenome of the semiterrestrial crab *Chiromantes neglectum* was sequenced. It contained the entire set of 37 genes. The gene order was basically identical to pancrustacean ground pattern, except for the*trnH* and *trnQ* genes. Phylogenetic inferences based on protein-coding genes (PCGs) provide strong evidence that places *C. neglectum* within an intermingled ‘Grapsoidea & Ocypodoidea’ clade.

The complete mitogenome of the semiterrestrial crab *Chiromantes neglectum* (De Man 1887) was sequenced based on next-generation sequencing method (Illumina HiSeq 2000), with assistant Sanger sequencing method. *C. neglectum* was collected from Chongming Island, Shanghai, China (31°43'56.031” N, 121°14'17.346” E). The voucher specimen (WCYN09) was preserved in 95% ethanol and deposited in Jiangsu Key Laboratory for Biodiversity and Biotechnology, College of Life Sciences, Nanjing Normal University.

The complete sequence of mitogenome for *C. neglectum* was determined to be 15,920 bp in length with 75.6% A + T content (supporting information, Figure S1), under GenBank accession number KX156954. It contained an entire set of 37 genes plus a main non-coding region (NCR). All 13 PCGs were AT-biased (74.8% on average), with the highest A + T content (86.2%) in *atp8* and the lowest (67.9%) in *cox1*. The *lrRNA* and *srRNA* was 1,337 bp and 825 bp in length, with an A + T content of 81.0% and 79.0%, respectively. All set of 22 tRNAs were ranging from 63 to 73 bp in length with typical clover-leaf structures. The main NCR, located between *srRNA* and *trnQ*, was 729 bp in length. In addition, notable intergenic non-coding nucleotides were detected with a total nucleotide of 406 (ranging from 1 to 211 nt; Table 1).

**Table 1.** Organization of the *Chiromantes neglectum* mitochondrial genome^a^.

Feature	From	To	Length (bp)	Codons start	Codons stop	IGN^b^
*cox1*	1	1535	1535	ATG	TA	0
*trnL_2_* (UUR)	1536	1601	66			6
*cox2*	1608	2295	688	ATG	T	−1
*trnK*	2295	2365	71			−1
*trnD*	2365	2428	64			0
*atp8*	2429	2587	159	ATG	TAA	−7
*atp6*	2581	3255	675	ATT	TAA	0
*cox3*	3256	4047	792	ATG	TAA	−1
*trnG*	4047	4111	65			0
*nad3*	4112	4462	351	ATT	TAA	4
*trnA*	4467	4532	64			8
*trnR*	4539	4604	66			2
*trnN*	4607	4674	68			3
*trnS_1_* (AGN)	4678	4744	67			1
*trnE*	4746	4811	66			4
*trnH*	4816	4878	63			2
*trnF*	4881	4945	65			7
*nad5*	4953	6680	1728	ATG	TAA	20
*nad4*	6701	8062	1362	ATG	TAG	−7
*nad4L*	8056	8358	303	ATG	TAA	9
*trnT*	8368	8433	66			0
*trnP*	8434	8500	67			8
*nad6*	8509	9005	497	ATA	TA	0
*cob*	9006	10140	1135	ATG	T	0
*trnS_2_* (UCN)	10,141	10,207	67			19
*nad1*	10,227	11,165	939	ATA	TAA	37
*trnL_1_* (CUN)	11,203	11,269	67			0
*lrRNA*	11,270	12,606	1337			0
*trnV*	12,607	12,679	73			0
*srRNA*	12,680	13,504	825			0
*NCR*	13,505	14,233	729			0
*trnQ*	14,234	14,302	69			211
*trnI*	14,514	14,580	67			63
*trnM*	14,644	14,713	70			0
*nad2*	14,714	15,721	1008	ATG	TAG	−2
*trnW*	15,720	15,788	69			2
*trnC*	15,791	15,854	64			0
*trnY*	15,855	15,920	66			0
Total		15,920	15,533			406/−19

^a^ The location of the 5′ and 3′ ends of all genes has not been confirmed experimentally; underline indicates the gene coded on the opposite strand. PCG is labeled with three-letter code; tRNA gene is given as uppercase letter code, except *trnL_1_* (CUN), *trnL*_2_ (UUR), *trnS_1_* (AGN) and *trnS_2_* (UCN).

^b^ IGN: Intergenic nucleotide. Positive number represents the size of t intergenic spacer separating two adjacent genes; negative number indicates that adjacent genes overlap.

Gene order of *C. neglectum* mitogenome was identical to pancrustacean ground pattern (Lavrov et al. 2004), except forthe *trnH* and *trnQ* genes (supporting information, Figure S1). Combining sequences of 13 PCGs from 25 brachyurans, both maximum likelihood and Bayesian analyses place the *C. neglectum* within an intermingled ‘Grapsoidea & Ocypodoidea’ clade (Figure 1). It confirm the results of previous analyses (Schubart et al. 2006; Tsang et al. 2014) that monophyles of these two superfamilies were negative in their current compositions. In addition, the heterotreme crabs appear paraphyletic in our analyses, with potamoid taxa being more closely related to thoracotreme crabs than to the remaining heterotremes. This confirms the result obtained from similar dataset by Ji et al. (2014). Whereas phylogenetic trees of Tsang et al. (2014) suggested that freshwater crabs align with Heterotremata rather than Thoracotremata. This difference in tree topologies may result from taxon sampling unevenly (Sheffield et al. 2009). Wider taxonomic sampling from divergent lineages of Potamoidea will be required in future studies.

**Figure 1.**
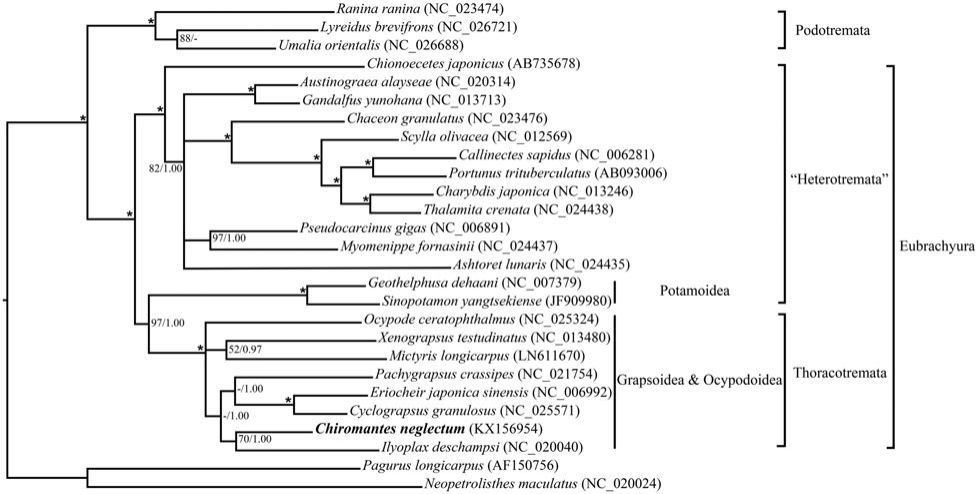
Phylogenetic relationship derived for brachyurans using maximum likelihood (ML) and Bayesian inference (BI) analyses using nucleotide sequences of 13 PCGs. Models of nucleotide evolution were chosen by the Bayesian information criterion (BIC) (www.robertlanfear.com/partitionfinder) (Lanfear et al. 2012). Phylogenetic trees were generated from maximum-likelihood analysis (RAxML; Stamatakis, 2014) under the GTR + G model and Bayesian inference (MrBayes; Huelsenbeck & Ronquist 2001) under the GTR + I + G model. Branch lengths and topologies came from ML analysis. Values at the branches represent BP (Bootstrap value)/BPP (Bayesian posterior probability). 100/1.00 is denoted by the asterisk. The horizontal line stands for BP under 50 or BPP under 0.9.
